# Epidemiology of food allergy in European infants

**DOI:** 10.1186/2045-7022-1-S1-S4

**Published:** 2011-08-12

**Authors:** Kirsten Beyer

**Affiliations:** 1 Charité, Klinik für Pädiatrie m.S. Pneumologie und Immunologie, Berlin, Germany

## 

The prevalence and causes of food allergy in Europe is still unclear. Individual studies show very different data. Within the EU-funded collaborative translational research project EuroPrevall, a meta-analysis was performed to assess the prevalence of food allergy. The foods assessed were cow’s milk, hen’s egg, peanut, fish, shellfish, and an overall estimate of food allergy. The prevalence of self-reported food allergy was ranging from 3% to 35% for any food and was very high compared with objective measures. Interestingly, there was marked heterogeneity between studies regardless of type of assessment or food item considered. Whether this reflects real differences or is a problem of different study designs could not be answered. In order to obtain comparable prevalence data for food allergy in Europe a multi-centre birth cohort study was started within EuroPrevall in 2005. A total of over 12,000 newborns from nine countries of four climatic regions across Europe were recruited. Standardised telephone interviews were scheduled at birth, 12, 24 and 30 months. In addition, parents were asked to immediately report to the study centres in case of the development of an atopic disease such as atopic dermatitis or about possible allergic reactions to food at any time. All children with atopic dermatitis that required treatment or symptoms possibly caused by food allergy were invited for standardised clinical evaluations including the diagnostic gold standard, double-blind placebo-controlled food challenge tests. Looking at the patterns of food allergy in these infants and young children we found that hen’s egg, cow’s milk, peanut and wheat were the most frequent food allergens in Europe. Interestingly, we saw a marked heterogeneity for some allergens between different countries despite the usage of the same study protocols and close monitoring. This finding might be important for future risk assessment in food allergy.

